# DFT investigation on the application of pure and doped X_12_N_12_ (X = B and Al) fullerene-like nano-cages toward the adsorption of temozolomide

**DOI:** 10.1098/rsos.211650

**Published:** 2022-04-06

**Authors:** Brice Laure Ndjopme Wandji, Aymard Didier Tamafo Fouegue, Nyiang Kennet Nkungli, Rahman Abdoul Ntieche, Abdoul Wahabou

**Affiliations:** ^1^ Department of Chemistry, Faculty of Science, The University of Maroua, PO Box 814, Maroua, Cameroon; ^2^ Department of Chemistry, Higher Teacher Training College Bertoua, The University of Bertoua, PO Box 652, Bertoua, Cameroon; ^3^ Department of Chemistry, Faculty of Science, The University of Bamenda, PO Box 39, Bambili-Bamenda, Cameroon

**Keywords:** temozolomide, fullerene-like nano-cages, DFT, adsorption

## Abstract

The sensitivity of pure and doped X_12_N_12_ (X = B and Al) fullerene-like nano-cages (FLNs) toward the anti-cancer drug temozolomide (TMZ) is probed herein at DFT/M06-2X-D3/6-311G(d,p) theoretical level in both gas phase and water. A noticeable affinity of the FLNs toward TMZ was observed along with the negative gas-phase adsorption energies −1.37 and −2.09 eV for the most stable configurations of pure B_12_N_12_ and Al_12_N_12_ pristines, respectively. Considerable charge transfer from TMZ to the FLNs was also revealed via NBO analysis and the Hirshfeld atomic charges, making the dipole moment vector of the molecular complexes to be oriented from the nano-cages to the TMZ moiety. Furthermore, a percentage decrease in the HOMO-LUMO energy gap (Δ*E*_g_) of 38.09 and 17.72% was obtained for the B_12_N_12_ and Al_12_N_12_ nano-cages, respectively. The percentage change in Δ*E*_g_ was found to be reduced upon doping and solvation of the FLNs. Finally, a recovery time in vacuum ultraviolet light of 1.06 s is found for the complex with pure B_12_N_12_, which in addition to the above-mentioned parameters make this boron nitride cage the best sensor for TMZ, among the FLNs considered in the present work.

## 1. Introduction

Temozolomide (TMZ) (marketed under the trademarks temodar or temodal) is an oral chemotherapeutic agent approved for the treatment of anaplastic astrocytoma and gliomas (the most common and most malignant primary tumours of the central nervous system). Malignant gliomas diffusive infiltration into adjacent brain parenchyma and resistance to cell death [1]. TMZ is spontaneously converted into the active alkylation metabolite (methyl-triazene-1-yl)-imidazole-4-carboxamide (MTIC) under physiological conditions. The commonest side effects of TMZ include nausea, diarrhea, vomiting, headaches, constipation, taste changes and rashes. The less common side effects are hair loss (including eyebrows and eyelashes), dizziness, shortness of breath, infertility (depending on the combination of medicines and the dose that is given) [2]. TMZ has also been reported to affect CD4 + T cells, leading to clinically significant opportunistic infections like *Pneumocystis carinii*, *Aspergillus pneumoniae*, herpes simplex, herpes zoster, hepatitis B virus etc. [1–5]. It is worthy of note that patients suffering from glioma may easily die from infectious complications. Owing to the wide therapeutic scale and effects of TMZ, Mariano and co-workers have reported several analytical methods for its detection [6]. In this regard, nanotechnologies have been of paramount importance in recent decades.

Experimental and theoretical chemistry methods are widely used in probing the sensing ability of nanoparticles (NPs) toward various classes of chemicals. Organic NPs (like lipids and micelles), inorganic NPs (like metal oxide, quantum dots and fullerenes) or a hybrid of these components are the common classes of NPs used for sensing and drug delivery. Several studies have been published on carbonic and non-carbonic nanostructures such as nano-sheets [7–9], fullerene-like nano-cages (FLN) [10,11] and nano-tubes [12,13], due to their excellent physical, chemical and surface characteristic properties. Among these NPs, FLNs are recently receiving considerable attention. They have been found to be excellent gas sensors [14–16]. One of the most prominent fields of application of the FLNs is their use as drug sensors, as well as carriers for drug delivery. In that regard, the adsorption of anti-cancer drugs, including thioguanine isomers [11], melphalan [17], Zolinza drug [10], 5-fluorouracil [18], exemestane [19] and Celecoxib [20] at the external surface of FLNs has been reported. The aforementioned works have highlighted the potential of FLNs as a medium for anti-cancer drug delivery.

The main objective of this work was to probe the adsorption of the anti-cancer drug TMZ onto the external surface of pure and doped X_12_N_12_ (X = B and Al) using the density functional theory (DFT) computational chemistry method. Interestingly, DFT calculations have been used to study the adsorption of TMZ onto the surface of iron-oxide (Fe_3_O_4_) nanoparticles via an interaction involving the oxygen and nitrogen atoms of the former, and the Fe^2+^ and/or Fe^3+^ ion of the latter [21]. Furthermore, Zhu and co-workers have recently shown, via the DFT/B3LYP/6-31G (d) method, that pristine BC_3_NT is a promising drug deliverer for TMZ [8]. Nevertheless, B_12_N_12_ and Al_12_N_12_ have been proven to be the most stable FLN structures, which have been synthesized by Oku *et al.* [22] and Liu *et al.* [23], respectively. These compounds possess unique physico-chemical properties such as high thermal conductivity, chemical stability, wide band gap and oxidation resistance [24]. Owing to the exceptional results obtained for the delivery of many anti-cancer drugs by B_12_N_12_ and Al_12_N_12_, the adsorption of TMZ at the surface of these nano-cages is investigated herein in order to propose a new drug delivery system for TMZ. The adsorbent–adsorbate interactions have been extensively described using the quantum theory of atoms in molecules (QTAIM) and natural bond orbital (NBO) analysis. The effects of adsorption on the electronic properties (such as molecular orbitals energy and distribution and electronic spectra) of the nano-cages have also been evaluated. The calculations have been performed using DFT methods in gas phase and water. The findings of this research endeavour can be useful for designing an appropriate and novel carrier for the detection, as well as the delivery, of TMZ.

## 2. Computational details

All molecular structures considered here have been relaxed at DFT/M06-2X/6-311G(d,p) [25–27] level of theory using the Gaussian 09 package [28]. The reliability of the M06-2X hybrid functional for such calculations has long been proven since it accurately estimates structural, energetic and electronic properties [29–31]. The Gordon dispersion GD3 correction was applied in order to better describe the non-covalent interactions (NCI) in the molecules studied [32]. Harmonic vibrational frequencies have been calculated at the same computational level after geometry relaxation, and their analysis showed that all structures corresponded to minima on their potential energy surfaces. The adsorption ability of the nano-cages toward TMZ was evaluated through adsorption energies (Δ*E*_Ads_), enthalpies (Δ*H*) and free energy changes (Δ*G*), calculated as shown in equations (2.1)–(2.3). *E*_FLN–TMZ_ is the total energy of the complex formed between the nano-cage and TMZ, whereas *E*_FLN_ and *E*_TMZ_ are the total energies of the FLNs and TMZ, respectively.

2.1 $$E_{\mathrm{Ads}}=E_{\mathrm{FLN}-\mathrm{TMZ}}-E_{\mathrm{FLN}}-E_{\mathrm{TMZ}},$$

2.2 $$\Delta H=H_{\mathrm{FLN}-\mathrm{TMZ}}-H_{\mathrm{FLN}}-H_{\mathrm{TMZ}}$$

2.3 $$\;\mathrm{and}\;\Delta G=G_{\mathrm{FLN}-\mathrm{TMZ}}-G_{\mathrm{FLN}}-G_{\mathrm{TMZ}}.$$

The energy and distribution of the frontier molecular orbitals of the investigated molecular systems were also studied in a bid to gain more insight into their stability. The NBO analysis at the above-mentioned level of theory has been performed to deepen our understanding of the FLN–TMZ interactions. This was further buttressed via QTAIM and NCI index analyses performed using the multiwfn 3.8 software [33]. The electronic transition spectra were calculated using the TD-DFT method at the M06-2X-D3/6-311G(d,p) theoretical level. Calculations in water medium were performed using the SMD solvation method [34].

## 3. Results and discussion

### 3.1. Geometric and energetic parameters

**Figure 1.**
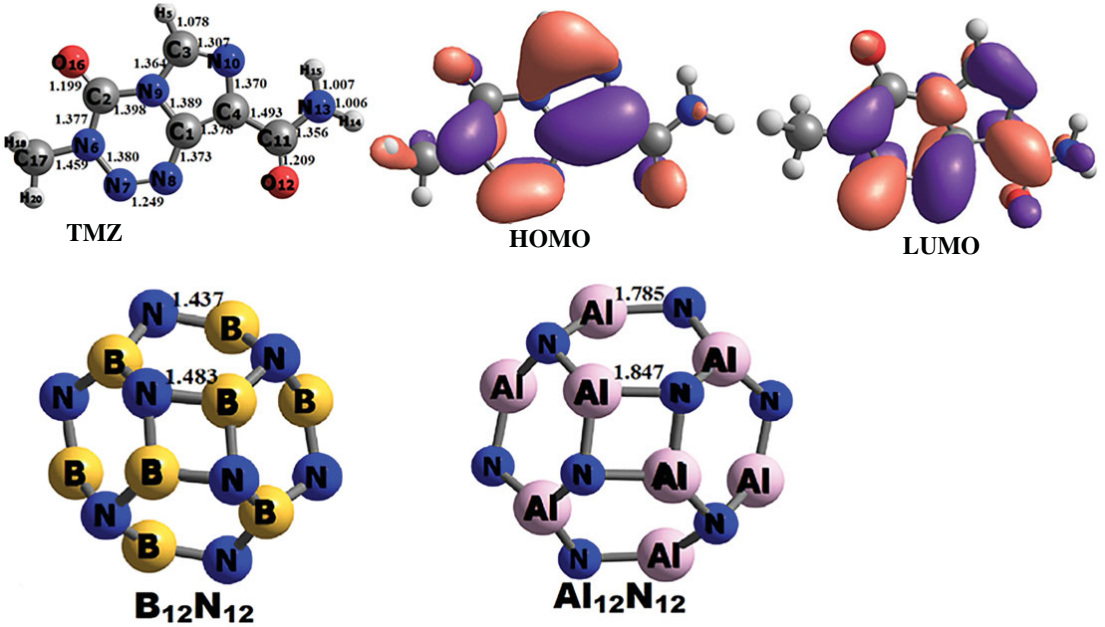
Gas-phase relaxed structures of TMZ, B_12_N_12_ and Al_12_N_12_.

**Figure 2.**
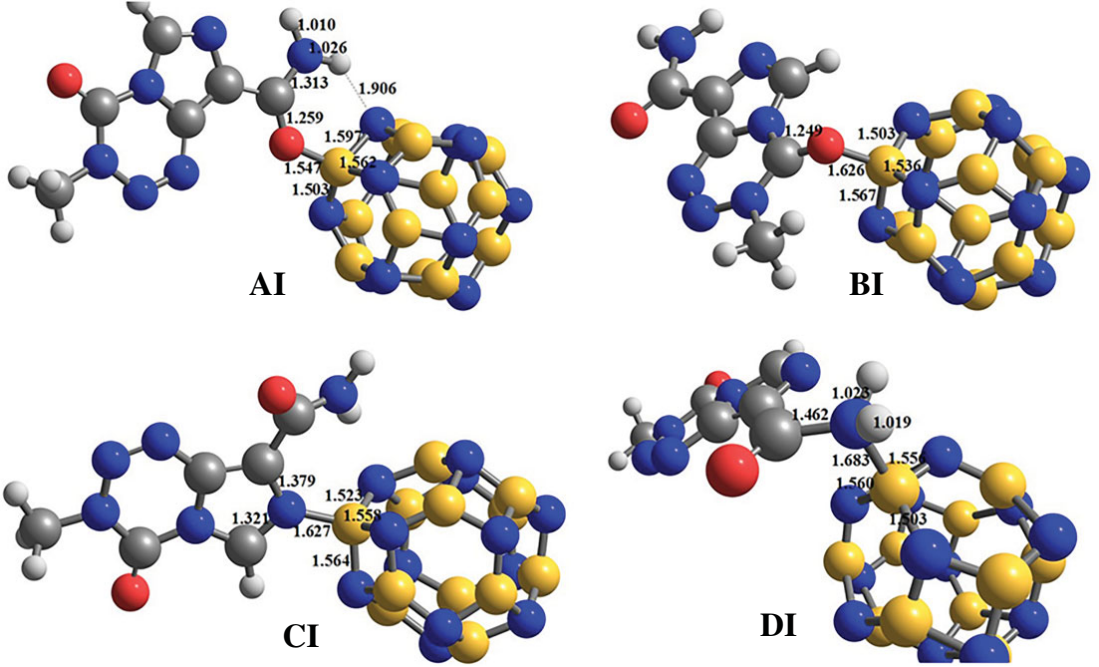
Gas-phase optimized structures of complexes formed between B_12_N_12_ and TMZ.

The optimized structures of TMZ, B_12_N_12_ and Al_12_N_12_ are depicted in figure 1, alongside the distribution of the FMOs of TMZ. The geometric parameters obtained herein from the optimized structures of pure B_12_N_12_ (B-N bond lengths and angles) and Al_12_N_12_ (Al-N bond lengths and angles) are in perfect agreement with those reported in the literature [35–37]. The structure obtained from the relaxation of TMZ is displayed in figure 1, along with the interatomic distances in gas phase. In order to locate the sites of TMZ exhibiting the greatest Lewis basic character, the Hirshfeld atomic charges of its atoms have been calculated. According to the results, O_12_ (−0.36 e), O_16_ (−0.32 e), N_10_ (−0.19 e) and N_13_ (−0.16 e) carry the most negative charges. The molecular electrostatic potential (MEP) maps of TMZ depicted in electronic supplementary material, figure 1S corroborates this finding. Indeed, the region near the vicinity of O_12_ and O_16_, coloured in red in electronic supplementary material, figure 1S indicates the high nucleophile character of these atoms. The yellow coloured of the MEP map around N_10_ of TMZ also shows an electron-rich site. These atoms are, therefore, predicted to be the most prominent to form Lewis-type acid-base interactions with the boron and aluminium atoms of the FLNs. This observation is in good agreement with the findings of Harris [21] who predicted, via frontier molecular orbitals analysis, that these atoms are the most reactive sites of TMZ. Therefore, the ability of the pure FLNs considered in the present work to adsorb TMZ at their external surface has been evaluated by simulating the adsorbent–adsorbate interactions including the foregoing atoms. The relaxed structures of the complexes simulated at the M06-2X-D3/6-311G(d,p) level in the case of B_12_N_12_ are depicted in figure 2, whereas those of Al_12_N_12_ are presented in electronic supplementary material, figure S1. The energetic parameters (*E*_Ads_, Δ*H* and Δ*G*) describing the adsorption process are summarized in table 1. In order to facilitate the discussion of the results, the complexes are denoted A, B, C and D corresponding to O_12_, O_16_, N_10_ and N_13_ respectively. The complexes involving the B_12_N_12_ and Al_12_N_12_ FLNs are distinguished by the use of the roman numerals I and II, respectively, after the above-mentioned letters (figure 2 and electronic supplementary material, figure 2S).

**Table 1.** Values of E_Ads_ (eV) ΔH (kcal mol^−1^) and ΔG (kcal mol^−1^) obtained at M06-2X-D3/6-311G(d,p) level.

	gas			water		
	*E*_Ads_	Δ*H*	Δ*G*	*E*_Ads_	Δ*H*	Δ*G*
AI	−1.37	−30.03	−16.43	−1.16	−27.23	−13.20
BI	−0.65	−14.01	−0.82	—	—	—
CI	−1.29	−28.83	−14.01	−1.04	−24.55	−10.14
DI	−0.92	−19.53	−6.42	—	—	—
AI_Al	−2.80	−63.01	−49.78	−2.48	−57.87	−44.59
CI_Al	−2.35	−52.53	−39.27	−2.07	−48.47	−35.08
AII	−2.09	−47.27	−33.88	−1.83	−42.88	−28.89
BII	−1.39	−30.76	−17.91	—	—	—
CII	−1.78	−39.74	−26.44	−1.48	−34.68	−21.58
DII	−1.33	−29.06	−16.40	—	—	—
AII_B	−0.36	−7.81	5.03	−0.11	−1.74	12.06
CII_B	−0.36	−7.22	5.99	−0.02	0.40	14.51

From the values in table 1, all complexes comprising B_12_N_12_ are interestingly found to be stable, since they are characterized by negative values of *E*_Ads_. The adsorption energies follow the trend: AI < CI < DI < BI. Accordingly, AI is the most stable of all the complexes studied. Zhu and co-workers studied the adsorption of TMZ on pristine BC_3_NT by linking the former to the adsorbent via its two oxygen atoms [8]. The adsorption energies found at the B3LYP/6-31G(d) level are, respectively, −18.84 kcal mol^−1^ (−0.8717 eV) when BC_3_NT is linked to O_12_ and −16.73 kcal mol^−1^ (−0.7256 eV) when it is linked to O_16_. Therefore, the B_12_N_12_ FLN is a better adsorbent of TMZ than BC_3_NT, since the most stable complex herein has a lower adsorption energy (−1.3697 eV) than that of Zhu *et al.* [8]. However, in the case of O_16_, our *E*_Ads_ value (−0.6564 eV) is similar to that of Zhu *et al*. The gas-phase enthalpy and Gibbs free energy change are also negative, suggesting exergonic and exothermic processes. The values of these thermodynamic parameters are found to follow the same trend as the adsorption energy. Increment in the bond lengths around the adsorption sites of both TMZ and the B_12_N_12_ FLN are observed upon adsorption. In the case of AI, the interatomic distance between C_11_ and O_12_ increases from 1.209 to 1.259 Å, and the B-N bond lengths of the nano-cage are found to increase from 1.483 to 1.562 and 1.597 Å (in the four-membered ring (4-MR) of the nano-cage), as well as from 1.437 to 1.503 Å in the 6-MR. The increment in the C_2_ = O_16_ bond length in BI is also 0.050 Å (from 1.199 to 1.249 Å). Additionally in BI, the B-N bond lengths of the nano-cage increased from 1.483 to 1.536 or 1.567 Å in the 4-MRs, and from 1.437 to 1.503 Å in the 6-MR. Similar observations were made in the cases of CI and DI. Moreover, the lengths of the B-O/B-N bonds linking the nano-cage (B_12_N_12_) to TMZ are found to be 1.547, 1.626, 1.627 and 1.683 Å respectively in AI, BI, CI and DI. Consequently, the most stable complex (AI) is characterized by the smallest adsorbent–adsorbate interatomic interaction. Zhu and co-workers obtained B-O_12_ and B-O_16_ interatomic distances of 1.82 and 1.91 Å, respectively, in their previous work [8]. These values are obviously greater than those obtained from the B_12_N_12_ nano-cages in the present work (AI and BI).

Table 1 also shows that all of the four complexes of Al_12_N_12_ (denoted XII, where X = A, B, C and D) have negative adsorption energy, Δ*H* and Δ*G* values in gas-phase. Moreover, these energetic parameters are more negative for each of these molecular systems than those of their respective counterparts comprising B_12_N_12_ as the adsorbent. However, these parameters are found to increase in the same order as those for the boron-nitride nano-cage. In studying the adsorption of pyrrole on some fullerene-like nano-cages, Rad and Ayub also found that the adsorption energy of the complex involving Al_12_N_12_ was more negative than that of B_12_N_12_ [38]. A similar observation has been made by other researchers [24,39,40]. The values of the bond lengths around the adsorption sites of AII, BII, CII and DII are provided in electronic supplementary material, figure S1. The Al-O/Al-N interatomic distances within these complexes have the values 1.870, 1.918, 2.009 and 2.066 Å, which are greater than those obtained from the complexes of B_12_N_12_. In this case, the complexes involving O_12_ (AII) which is the most stable, also features the lowest adsorbent–adsorbate interatomic interaction. The C_11_=O_12_, N_10_-C_3_ and N_10_-C_4_ bond lengths of TMZ upon adsorption to the external surface of Al_12_N_12_ FLN (AII and CII), are similar to those of AI and CI. However, the C_2_=O_16_ interatomic distance in BII (1.241 Å) is shorter than that in BI (1.249 Å). A similar observation is made with regard to the C_11_-N_13_ bond length in BL, which is shorter in DII (1.441 Å) than in DI (1.462 Å).

### 3.2. Effects of doping and solvation

Doping is a routinely used method for improving the sensing and adsorption ability of FLNs. In this perspective, Bahrami and co-workers reported the good adsorption ability of Al-doped B_11_N_12_ FLN toward amphetamine [41]. Similar results were published for the adsorption of aspirin by Vessally *et al*. [35]. Moreover, the B_11_GaN_12_ nano-cluster was also reported to be a better sensor for CO than pure B_12_N_12_ [14]. In the present research endeavour, the ability of Al-doped B_12_N_12_ and B-doped Al_12_N_12_ to adsorb TMZ is probed. In this analysis, only the two most stable structures obtained with the pure nano-clusters (AI, CI, AII and CII) are considered. The optimized structures of the doped FLNs and their complexes are displayed in electronic supplementary material, figure 3S alongside some of their interatomic distances around the adsorption sites. The complexes involving the Al-doped B_12_N_12_ are dubbed AI-Al and CI-Al, whereas those of the BAl_11_N_12_ are represented by AII-B and CII-B. The length of the Al-N bond in the relaxed structure of AlB_11_N_12_ is found to be 1.785 and 1.824 Å, values which perfectly agree with those obtained by Vessally *et al*. [35] The B-N bond lengths in the BAl_11_N_12_ nano-cluster are 1.515 Å (in the 4-MR) and 1.447 Å (in the 6-MR).

The gas-phase values of *E*_Ads_, Δ*H* and Δ*G* describing the adsorption of TMZ at the surface of the doped nano-clusters are summarized in table 1. It turns out from the table that all the values of the adsorption energy for both doped FLN are negative, but those involving the O_12_-atom of TMZ are the lowest. Accordingly, AI-Al and AII-B are more stable than CI-Al and CII-B, respectively. Furthermore, the values of *E*_Ads_ obtained with AlB_11_N_12_ are more negative than those of pure B_12_N_12_, which interestingly reveals an improvement in the adsorption ability of the doped nano-cages. In addition, the adsorption reactions involving AlB_11_N_12_ are more exothermic and more exergonic than those of pure B_12_N_12_. However, unlike the non-doped BN nano-cage, the doping of Al_12_N_12_ leads to an increment in the *E*_Ads_, Δ*H* and Δ*G* values (yielding more positive values). Although exothermic, the gas-phase adsorption of TMZ at the external surface of the B-doped Al_12_N_12_ FLN is non-spontaneous under standard conditions.

Adsorption at the external surface of the Al-doped B_12_N_12_ leads to a greater change in the C_11_=O_12_ bond length of TMZ than is the case with adsorption at the surface of pure B_12_N_12_. Indeed, the length of these bonds increase by 0.058 Å (i.e. from 1.209 to 1.267 Å) in AI_Al, which is slightly greater than 0.050 Å in the case of AI. An opposite trend is observed between AII and AII_B, implying that the length of the C_11_=O_12_ bond in the first complex (1.260 Å) is greater than that in the second (1.248 Å). Furthermore, the discrepancies in the values of the C_3_-N_10_ and C_4_-N_10_ bond lengths between the complexes comprising the pure and doped FLN are negligible.

### 3.3. Topological analysis

In theoretical chemistry, the nature and strength of non-covalent interactions are frequently characterized via a set of methods known as topological analysis techniques, of which the quantum theory of atoms in molecules (QTAIM) is the most popular. QTAIM topological analysis is based on the partitioning of a molecular space into a set of interconnected critical points (CPs) [42]. A CP is a space point where the first derivative of the electron density function *ρ*(**r**) is null. Therefore, the philosophy of the Bader's QTAIM approach is to define bonds using the electron density. According to that theory, an interaction between two atoms is identified by the presence of a bond path between them. The presence of a bond path or a bond critical point (BCP) between two atoms confirms the existence of a covalent or non-covalent interaction between the atoms. Within the QTAIM framework, covalent and non-covalent interactions can be distinguished as follows [43]:

– Shared interactions for which the Laplacian of the electron density ∇²*ρ*(**r**) is negative at the BCP (3, −1) are classified as covalent bonds.
– Closed shell interactions for which ∇²*ρ*(**r**) greater than 0 are ionic or Van der Waals (VdW) interactions.

In the present work, the Bader's QTAIM has been used as implemented in the multiwfn 3.8 package, to characterize FLN-TMZ interactions in all the complexes under investigation. For that purpose, in addition to *ρ*(**r**) and ∇²*ρ*(**r**), the identified hydrogen bonds (HBs) were also characterized by their energy, approximated as half of the potential energy density v(**r**) at the BCPs. The sum (denoted h(**r**)) of the kinetic energy density at the BCP, g(**r**), and the potential energy density, v(**r**), is also useful in determining the nature of HBs. Negative values of h(**r**) are associated with strong HBs, while positive values are associated with both intermediate and weak HBs.

The Bader's topological analysis in this work has been complemented by the visual analysis of non-covalent interactions using the reduced gradient density (RDG) to identify non-covalent interactions (NCIs) [44]. This method uses coloured iso-surfaces to distinguish between different types of NCIs. Strong interactions like conventional HBs are characterized by blue-coloured iso-surfaces, whereas the green-coloured surfaces indicate attractive VDW interactions. Red-coloured iso-surfaces describe repulsive interactions.

**Figure 3.**
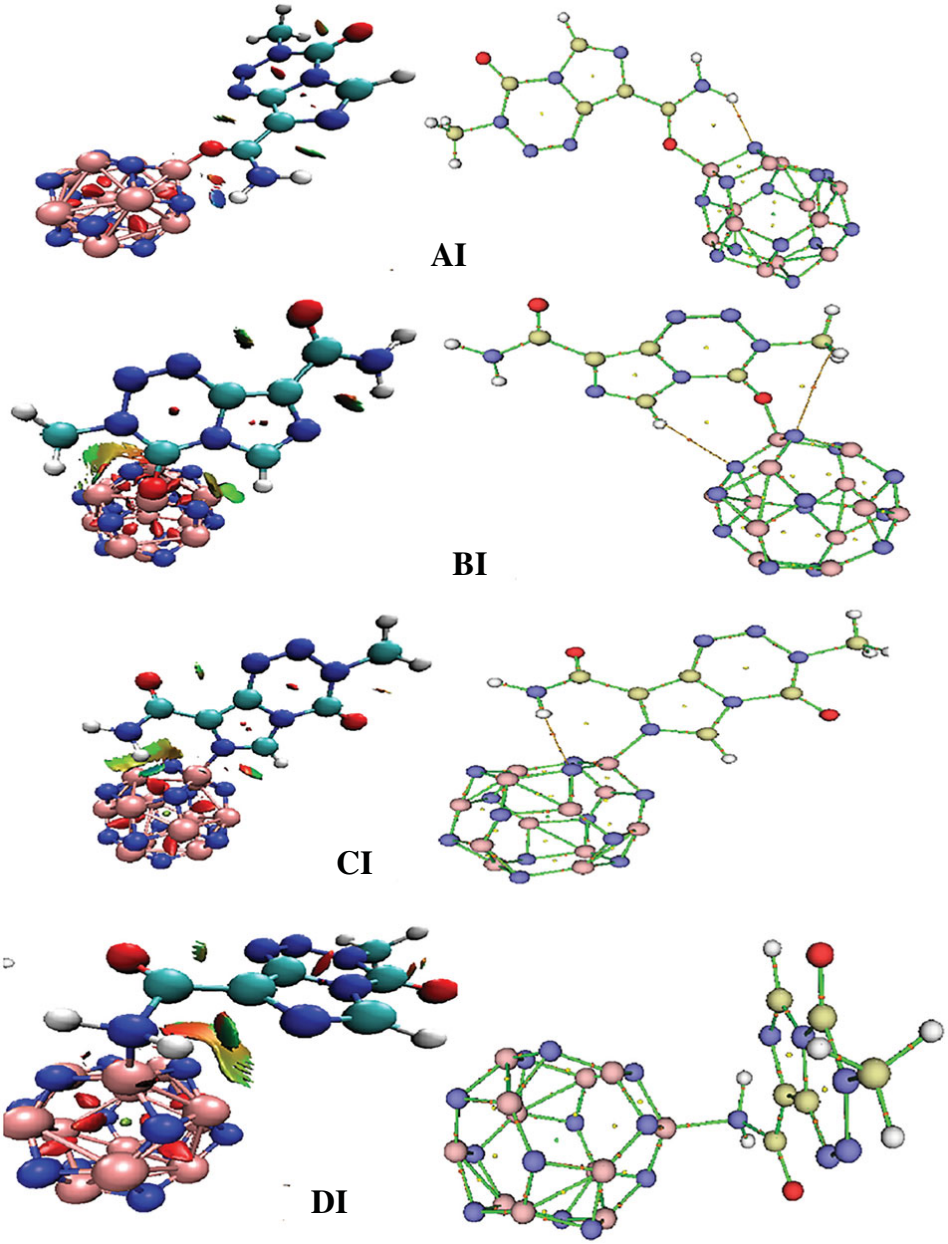
Molecular diagrams and visualization of NCI of AI, BI, CI and DI obtained from their gas phase optimized structures.

The molecular diagrams of AI, BI, CI and DI are displayed in figure 3, along with their respective NCI iso-surfaces. Some topological parameters describing the interactions between the B_12_N_12_ FLNs and TMZ are summarized in table 2. A close inspection of figure 3 reveals that apart from DI, all complexes involving the boron nitride FLN have, in addition to the B-O/B-N bonds, HB interactions. These HBs apparently contribute significantly to the stability of the complexes. A closer examination of table 2 reveals that the absolute value of the B-O/B-N energies increases as in the order: BI < DI < CI < AI. Interestingly, this trend shows that the stability of the complexes is directly proportional to their B-O/B-N interaction, since the most stable complex AI (with the lowest *E*_Ads_ value and the most spontaneous formation reaction) exhibits the strongest B–O interaction (−81.33 kcal mol^−1^). Similarly, the least stable complex has the lowest B-O interaction energy. Moreover, table 2 shows that among these complexes, only AI possesses a hydrogen bond that satisfies the intermediate HB conditions, which according to Rozas *et al*. [45] are positive values of ∇²*ρ*(**r**) (0.1040 a.u.) and negative values of h(**r**) (−0.0002 a.u.). This observation is confirmed by the blue-coloured iso-surface in NCI plot of AI. The strength of this HB with energy −8.30 kcal mol^−1^ certainly increases the stability of AI. The NH … N and CH … N interactions in BI and CI rather have the characteristics of weak HBs, which are positive values of ∇²*ρ*(**r**) and h(**r**). The green-coloured iso-surface in their NCI diagrams confirm the attractive character of these interactions, which seemingly impact the stability of the molecular systems.

**Table 2.** Some relevant topological parameters obtained from the optimized structures of the compounds under investigation.

		*ρ*(r)	∇²*ρ*(r)	h(**r**)	*E* (kcal mol^−1^)
AI	B-O_12_	0.1156	0.5181	−0.0649	−81.33
	N_13_H … N	0.0329	0.1040	−0.0002	−8.30
BI	B-O_16_	0.0922	0.3632	−0.0501	−59.95
	C_17_H … N	0.0068	0.0229	0.0008	−1.26
	C_3_H … N	0.0105	0.0323	0.0012	−1.79
CI	B-N_10_	0.1156	0.3249	−0.0789	−75.00
	N_13_H … N	0.0203	0.0746	0.0025	−4.28
DI	B-N_13_	0.1042	0.2390	−0.0712	−63.42
AI_Al	B-O_12_	0.0710	0.5428	−0.0067	−38.34
	N_13_H … N	0.0328	0.0965	−0.0008	−8.08
CI_Al	B-N_10_	0.0632	0.3723	−0.0016	−30.21
	N_13_H … N	0.0231	0.0797	0.0019	−5.06
AII	Al-O_12_	0.0630	0.4652	0.0068	−32.22
	N_13_H … N	0.0507	0.1028	−0.0104	−14.60
BII	Al-O_16_	0.0529	0.3759	0.0064	−25.43
	C_17_H … N	0.0104	0.0280	0.0007	−1.75
	C_3_H … N	0.0144	0.0443	0.0014	−2.60
CII	Al-N_10_	0.0541	0.3088	−0.0003	−24.38
	N_13_H … N	0.0315	0.0938	−0.0005	−7.69
	C_3_H … N	0.0156	0.0530	0.0017	−3.09
DII	Al-N_13_	0.0484	0.2561	−0.0007	−20.54
	C_4_ … N	0.0130	0.0402	0.0009	−2.58
AII_B	B-O_12_	0.0912	0.3296	−0.0507	−57.66
	N_13_H … N	0.0460	0.1103	−0.0069	−13.01
CII_B	B-N_10_	0.0895	0.2057	−0.0575	−51.19
	N_13_H … N	0.0223	0.0704	0.0016	−4.53
	N_13_ … N	0.0082	0.0254	0.0008	−1.52

The molecular diagrams of XII (X = A, B, C and D) complexes as well as their visual NCIs are depicted in electronic supplementary material, figure 4S. The topological parameters describing the Al_12_N_12_-TMZ interactions are listed in table 2. As can be easily seen, electronic supplementary material, figure 4S reveals that in addition to the B-O/B-N bonds, the HBs in the XI (X = A, B, C and D) complexes are localized between the Al_12_N_12_ and TMZ moieties. However, CII and DII present a C_3_H … N and a C_4_ … N attractive interaction, respectively, that are not found in CI and DI. Analysis of table 2 shows that the B-O/B-N interactions have lower interaction energy values in the Al_12_N_12_-TMZ complexes than in the B_12_N_12_ ones. Nevertheless, the HBs and attractive VdW interactions in these complexes are stronger (with higher absolute values of interaction energies, as well as higher value of *ρ*(**r**)) than those of the B_12_N_12_ FLN under investigation. Furthermore, unlike the N_13_H … N HB of CI, that of CII is an intermediate type HB. This is corroborated by the blue-coloured iso-surface of the NCI diagram of CII that is contrary to the green colour in the case of CI.

It can be observed from table 2 that the values of electron density as well as those of the interaction energy of Al-O_12_ and Al-N_10_ bonds in Al-doped complexes (AI_Al and CI_Al) are lower than those obtained with pure B_12_N_12_ (B-O_12_ and B-N_10_). The reverse is observed when the *ρ*(**r**) and E values of Al_12_N_12_ and BAl_11_N_12_ are compared. However, the values of adsorption energy in the molecular systems involving direct interactions of TMZ with the boron atom were found in the previous section to be lower than those involving direct Al-TMZ interactions (Al_12_N_12_ and AlB_11_N_12_). This is indicative of the fact that the said interactions might not be playing a prominent role in stabilizing the molecular systems obtained via adsorption.

### 3.4. Natural bond orbital analysis

Natural bond orbital (NBO) allows the quantification of interaction of electron based on the Lewis theory. It uses the stabilization energy E^(2)^, which is estimated based on the second order perturbation theory, to predict the strength of Lewis-type donor-acceptor interactions that contribute to the electron delocalization from bonding (BD) or lone pair orbitals (LP) to anti-bonding orbitals (BD*) [46,47]. Large values of E^(2)^ indicate strong interactions. Several interactions in both the nano-cage and TMZ moieties have been observed from calculations of NBO parameters. In the present work, only the values of the stabilization energy E^(2)^ (greater than 10 kcal mol^−1^) describing the interactions between the FLNs and TMZ are reported (table 3) in order to deepen the description of the said interactions obtained from QTAIM and NCI analyses. It can be observed from table 3 that AI and BI are mainly stabilized by interactions between lone pairs from O_12_ or O_16_ to the anti-bonding orbitals involving the boron-atom through which the FLN links to TMZ as well as the N-atoms adjacent to it. In AI for instance, a strong electron delocalization from a LP of O_16_ to a σ*(B_8_-N_14_) with E^(2)^ value of 149.30 kcal mol^−1^ is observed. A similar observation is made in BI, with stabilizing energy of 144.14 kcal mol^−1^ corresponding to the LP(O_12_) → σ*(N_15_-B_18_) interaction. In addition to these interactions, AI and BI are also stabilized by *σ*(C_2_ = O_16_) → σ*(N_4_-B_18_) (E^(2)^ = 14.82 kcal mol^−1^) and *σ*(C_11_ = O_12_) → σ*(N_15_-B_18_) (E^(2)^ = 14.32 kcal mol^−1^) interactions, respectively. Moreover, the N_28_-H … N hydrogen bond previously observed in the molecular structure of AI is also confirmed to greatly contribute to the stabilization of the said complexes. Indeed, an interaction energy of 12.05 kcal mol^−1^ describing that HB bond is found from the NBO analysis. Interactions within the frameworks of FLN and TMZ are found to be of paramount importance to the stability of the CI and DI complexes, and only one major interaction involving both structures is obtained in CI. Specifically, this interaction involves the delocalization of electron density from the LP of one N-atoms of the FLN (adjacent to the B-atom of the cage linked to TMZ) to the anti-bonding orbital of the B-N_10_ bond enabling the interaction between the adsorbent and the adsorbate. Similarly, electron density delocalization from the LP of all three N-atoms neighbouring the B-atom of B_12_N_12_ bearing the TMZ structure to σ*(B-N_13_) (leading to the B_12_N_12_-TMZ complex) are observed in DI.

**Table 3.** Values of the second-order perturbation energy E^(2)^ calculated in gas phase.

	donor	acceptor	E^(2)^ (kcal mol^−1^)		donor	acceptor	E^(2)^ (kcal mol^−1^)
AI	LP(O_16_)	σ*(B_8_-N_15_)	18.94	AII	LP(O_16_)	σ*(Al_33_- N_8_)	44.37
	LP(O_16_)	σ*(B_8_-N_4_)	95.21		LP(O_16_)	σ*(Al_33_- N_8_)	13.56
	LP(O_16_)	σ*(B_8_-N_4_)	12.68		LP(O_16_)	σ*(Al_33_- N_7_)	21.03
	*σ*(C_2_-O_16_)	σ*(B_8_-N_4_)	14.82		LP(O_16_)	Ry*(Al_33_)	14.76
	LP(O_16_)	σ*(B_8_-N_14_)	149.30		LP(O_16_)	σ*(Al_33_- N_8_)	10.86
	LP(O_16_)	σ*(B_8_-N_14_)	10.44		*σ*(C_2_ = O_16_)	σ*(Al_33_- N_8_)	10.00
	LP(N) from FLN	σ*(N_28_-H_31_)	12.05		*σ*(Al_33_- N_7_)	σ*(N_28_-H_31_)	25.01
BI	LP(O_12_)	σ*(N_15_-B_18_)	144.14	BII	LP(O_12_)	σ*(Al_33_- N_7_)	14.78
	LP(O_12_)	σ*(N_15_-B_18_)	57.63		LP(O_12_)	σ*(Al_33_- N_7_)	24.02
	*σ*(C_11_-O_12_)	σ*(N_15_-B_18_)	14.32		LP(O_12_)	σ*(Al_33_- N_7_)	16.79
CI	LP(N_12_) from FLN	σ*(B-N_10_)	11.52	CII	LP(N_10_)	σ*(Al_33_- N_10_)	16.44
DI	LP(N_19_) from FLN	σ* (B – N_13_)	12.97		LP(N_10_)	σ*(Al_33_- N_4_)	38.74
	LP(N_12_) from FLN	σ* (B – N_13_)	13.66		LP(N_10_)	σ*(Al_33_- N_4_)	11.21
	LP(N_7_) from FLN	σ* (B – N_13_)	12.82	AII-B	*σ*(N_2_-B)	σ*(O_16_-B)	509.78
	*σ*(N_13_-B)	σ*(C-O_12_)	10.00		*σ*(N_7_-B)	σ*(O_16_-B)	23.01
AI-Al	LP(O_16_)	σ*(Al)	40.90		*σ*(N_2_-B)	σ*(O_16_-B)	19.36
	LP(O_16_)	σ*(Al)	15.80		*σ*(O_16_-B)	σ*(N_8_-B)	10.00
	LP(O_16_)	σ*(Al)	15.78		*σ*(O_16_-B)	σ*(N_8_-B)	11.41
	LP(O_16_)	σ*(Al)	56.57		*σ*(O_16_-B)	σ*(N_2_-B)	88.24
	LP(O_16_)	σ*(Al)	16.85	CII-B	*σ*(B- N_10_)	σ*(B-N_4_)	86.65
CI-Al	LP(N_10_)	σ*(Al)	44.91		*σ*(B- N_10_)	σ*(B-N_4_)	10.97
	LP(N_10_)	σ*(Al)	10.46				

Concerning the complexes of pure Al_12_N_12_, the interactions found in AII are similar to those in AI. The interaction from LP(O_16_) to σ*(Al_33_-N_8_) (with E^(2)^ = 44.37 kcal mol^−1^) is the main interaction involving both the cage and TMZ. The delocalization from *σ*(Al_33_-N_8_) to σ*(N_28_-H_31_) describing the HB with E^(2)^ value of 25.01 kcal mol^−1^ is observed. In the case of BII, the O-atom of TMZ (O_12_) that is linked to the FLN was found to delocalize electrons from its LP to the σ*(Al-N) of the cage, which stabilizes the complex. A similar observation is made in the case of CII. Finally, no major interaction involving both the nano-cage and TMZ is found in DII.

In the Al-doped complexes, electron transfer from the LP of O_16_ and N_10_ to the empty orbital of the doping Al-atom is considerably important in stabilizing the systems. The transitions with E^(2)^ values 56.57 and 59.13 kcal mol^−1^ are obtained in AI-Al and CI-Al, respectively. In the B-doped complexes, interactions involving the N_12_Al_11_B-TMZ bond (B-O_16_ and B-N_10_ of AII_B and CII_B, respectively) and the B-N anti-bonding orbital of the cage represent the principal electron delocalization interactions stabilizing the structures, as shown in table 3. An interesting interaction from *σ*(N-B) to σ*(O_16_-B) is revealed, with *E*^(2)^ value 509.78 kcal mol^−1^.

The absolute value of the Hirshfeld charges (HCs) of the B and N atoms in pure B_12_N_12_ is 0.33 e. The gas-phase HCs on the B and Al atoms linked to TMZ in all molecular complexes considered herein are summarized in electronic supplementary material, table 1S, along with those of the N-atoms adjacent to the B and Al atoms. For the molecular complexes involving the above-mentioned pristine, the HCs of the B-atom linked to TMZ are in the range 0.25–0.27 e, while those of the N-atoms bonded to the said B-atom are between −0.34 and −0.37 e. Therefore, the B and N atoms gain electron upon adsorption of TMZ at the external surface of B_12_N_12_. Moreover, it can be seen from electronic supplementary material, table 1S that the HCs of the O and N atoms of TMZ increase (as the charge becomes less negative) after its adsorption by the pure BN FLN. All these observations account for charge transfer between TMZ and the nano-cage. Similar remarks can be made with pure Al_12_N_12_ (though the change in the HCs of atoms is less significant than in the case of pure B_12_N_12_), as well as the doped FLN under investigation.

### 3.5. Electronic properties

**Figure 4.**
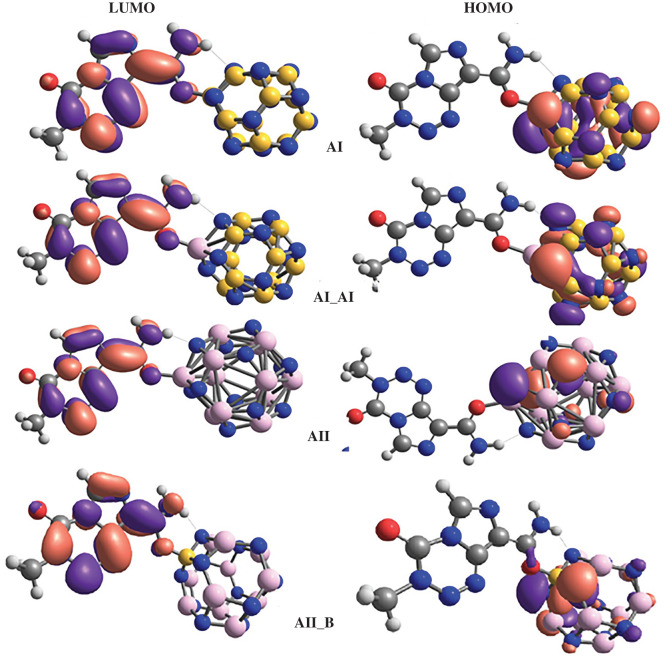
Distribution of FMO of the most stable configuration of molecular complexes obtained from the FLNs under investigation.

**Figure 5.**
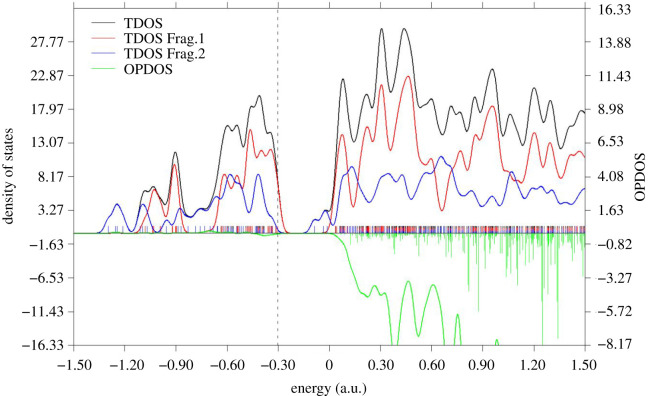
Partial density of state of TMZ adsorbed on the surface of B_12_N_12_ (Frag 1 = B_12_N_12_ and Frag 2 = TMZ). The dash line represents the HOMO level.

Electronic properties are key parameters in the description of the sensing ability of nano-cage materials. The HOMO and LUMO levels are of paramount importance in evaluating these parameters. The calculated gas and aqueous phases values of HOMO–LUMO gap (*E*_g_) and Fermi level (*E*_F_) of the molecular systems under investigation are summarized in table 4. The distributions of the FMOs of TMZ are also presented in figure 1, while those of AI, AI_Al, AII and AII_Al are presented in figure 4. It can be seen from figure 4 that the HOMO iso-surface distribution of all molecular complexes are nearly the same, and are exclusively centred on the N-atoms of the nano-cages. The LUMO iso-surfaces are similarly distributed but are mainly localized around the TMZ atoms. These findings corroborate the density of states (DOS) of these systems, which are also similar (reasons why only that of AI is presented in figure 5). Therefore, the adsorption of TMZ at the external surface of the pure and doped FLNs considered herein greatly modifies their FMOs, thereby altering their properties. Inspection of table 4 indicates an increment in the HOMO levels, as well as a change in the LUMO levels of all the FLNs upon adsorption of TMZ. This is a very important observation when probing the sensing ability of nanostructures via the theoretical chemistry methods. The *E*_g_ value of TMZ is 6.95 eV as evidenced in table 4. This parameter is, however, known to be very sensitive to the theoretical level. Zhu and co-workers published for TMZ, an *E*_g_ value of 3.97 eV at the B3LYP/6-31G(d) level of theory [8]. The gas-phase magnitudes of *E*_g_ obtained herein for B_12_N_12_ (9.44 eV) and Al_12_N_12_ (6.32 eV) perfectly agree with those obtained by Padash and co-workers at M062X/6-311G(d,p) level of theory [24]. Analysis of the values in table 4 also reveals that adsorption of TMZ at the external surface of pure B_12_N_12_ FLN leads to a huge reduction in its *E*_g_ value. The values of Δ*E*_g_(%) (calculated taking the FLNs as references) for the molecular complexes of that nano-cage increase as follows: CI < BI < DI < AI. The reference point for changes in HOMO and LUMO is the adsorbent (nano-cage here) [8,24]. It turns out that AI, which was found to be the most stable of these complexes has the greatest Δ*E*_g_(%) value of 38.09%. The magnitude of this value clearly shows that the FLN is very sensitive to TMZ, and can accordingly be efficiently applied as sensor for its detection. This result is consistent with the change in the Fermi levels, for which AI is still the molecular complex exhibiting the greatest change. In the case of pure Al_12_N_12_, the percentages of energy gap change upon adsorption of TMZ at the external surface of pure Al_12_N_12_ are significantly lower than those of B_12_N_12_. In that case, the greatest value of Δ*E*_g_(%) is 17.72, which is still obtained with AII. Furthermore, the change in the *E*_F_ values of the Al_12_N_12_ FLN is smaller than that of the B_12_N_12_ FLN. These observations clearly confirm the fact that pure B_12_N_12_ FLN is a better TMZ sensor than Al_12_N_12_.

**Table 4.** Energy of HOMO, LUMO, HOMO-LUMO gap (*E*_g_) in eV, percentage of the change of *E*_g_ after adsorption (Δ*E*_g_%), Fermi level (*E*_F_), dipole moment (Debye) and amount of charge transfer (Q(e)) between TMZ and the FLNs.

	gas							water					
	*E*_HOMO_	*E*_LUMO_	*E*_gap_	Δ*E*_g_ (%)	*E*_F_	*μ*	Q(e)	*E*_HOMO_	*E*_LUMO_	*E*_gap_	Δ*E*_g_ (%)	*E*_F_	*μ*
TMZ	−8.38	−1.43	6.95	—	−4.91	3.46	—	−8.19	−1.39	6.80	—	−4.79	5.22
B_12_N_12_	−9.45	−0,01	9.44	—	−4.73	0.00	—	−9.13	−0.47	8.66	—	−4.80	0.00
AI	−8.25	−2.39	5.85	38.09	−5.33	12.34	−0.13	−8.41	−1.76	6.65	23.21	−5.08	15.03
BI	−8.79	−2.44	6.34	32.91	−5.62	4.48	−0.40	—	—	—	—	—	—
CI	−8.69	−2.21	6.48	32.43	−5.45	7.85	−0.38	−8.55	−1.65	6.90	20.32	−5.10	10.67
DI	−8.52	−2.30	6.22	34.18	−5.41	8.66	−0.42	—	—	—	—	—	—
AlB_11_N_12_	−9.02	−2.40	6.62	—	−5.71	—	—	−8.81	−1.53	7.28	—	−5.17	5.54
AI_Al	−8.14	−2.67	5.46	17.52	−5.41	15.16	−0.38	−8.35	−1.86	6.49	10.85	−5.10	19.36
CI_Al	−8.38	−2.58	5.79	12.54	−5.49	10.61	−0.36	−8.37	−1.80	6.56	10.03	−5.09	15.31
Al_12_N_12_	−8.02	−1.70	6.32	—	−4.86		—	−8.09	−1.54	6.55	—	−4.81	
AII	−7.32	−2.12	5.20	17.72	−4.97	9.53	−0.26	−7.78	−1.69	6.08	7.18	−4.19	12.24
BII	−7.76	−2.17	5.59	11.55	−5.36	2.79	−0.33	—	—	—	—	—	—
CII	−7.74	−1.96	5.78	8.54	−4.85	*5.14*	−0.28	−7.84	−1.63	6.21	5.19	−4.73	7.59
DII	−7.60	−2.05	5.55	12.18	−4.83	5.82	−0.36	—	—	—	—	—	—
BAl_11_N_12_	−7.85	−1.69	6.16	—	−4.77	1.63	—	−7.97	−1.51	6.46	—	−4.74	2.92
AII_B	−7.08	−1.87	5.21	15.42	−4.48	7.40	−0.33	−7.50	−1.59	5.91	9.31	−4.54	9.39
CII_B	−7.63	−1.72	5.91	4.06	−4.68	4.32	−0.30	−7.61	−1.47	6.14	4.95	−4.54	6.97

The dipole moment of a molecular system strongly depends on the magnitude and separation of charges within the system, and is thus important in predicting their chemical reactivity. It is evident in table 4 that adsorption leads to huge increments in the dipole moment of the FLNs. In all cases, complex A is found to have the greatest value of the DM, indicating the strength of the covalent interaction between FLN and TMZ. This is corroborated by values of AIM and NBO parameters as well as the short adsorbent-TMZ interactions obtained in the molecular complexes investigated. The orientation of the DM vector in all the complexes shown (some of them depicted in electronic supplementary material, figure 5S) are from FLN to TMZ. The average values of the FLN HCs in all molecular complexes, as summarized in table 4, are all negative, implying those of TMZ are positive. This result affirms both the direction of the DM vector and the charge transfer from TMZ to the FLNs as highlighted previously.

Short recovery period (fast desorption) is a requirement for a suitable sensor. Therefore, too strong chemical interactions are not ideal for sensors, since such adsorption often leads to long recovery times which are not ideal for sensor applications [8]. The recovery time (*τ*) for complexes indexed as A (the most stable with respect to the different FLNs) was calculated as:

3.1 $$\tau =\upsilon_{0}^{-1}\exp\left(-\frac{\Delta G_{\mathrm{Ads}}}{\mathrm{kT}}\right)$$

*υ*_0_ is the frequency of the ultraviolet light in the vacuum (= 10^12^ s^−1^), used to extract the adsorbed molecule (TMZ), k is the Boltzmann's constant (1.99 kcal mol^−1^ K^−1^) and T is the room temperature (298.15 K). The recovery time thus depends on the exponential part (ΔG_Ads_). Values of *τ* summarized in table 5 clearly reveal that only AI with a recovery time of 1.06 s can be applied as sensor for the detection of TMZ. This result corroborates those observed on the analysis of electronic properties.

**Table 5.** Recovery time (*τ*) in vacuum ultraviolet light of the most stable molecular complexes obtained from the studied FLNs.

	AI	AI_Al	AII	AII_B
*τ* (s)	1.06	1.84 × 10^24^	4.73 × 10^24^	—

### 3.6. Electronic spectra analysis

**Figure 6.**
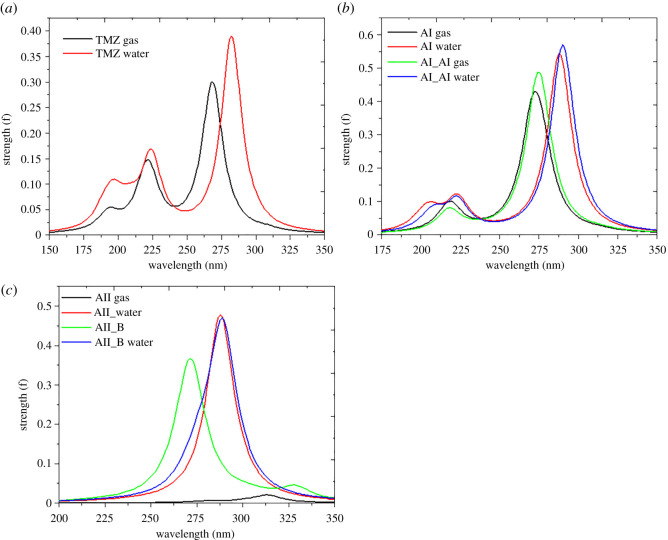
Electronic spectra of some compounds studied obtained at M06-2X-D3/6-311G(d,p) level of theory.

The TD-DFT/M06-2X-D3/6-311G(d,p) level of theory has been used to predict the absorption spectra of TMZ alongside its most stable complexes (AI, AII, AI_Al and AII_B) in both gas phase and aqueous environments. The results obtained are summarized in table 6, while the corresponding spectra diagrams are displayed in figure 6. Figure 6*a* shows three absorption maxima of the electronic spectrum of TMZ, the most intense of which appears at 268 nm in gas phase and at 282 nm in water. A red shift of the absorption maxima is evident upon solvation of TMZ. These maxima arise from electronic transitions originating from the HOMO to the LUMO of TMZ, which according to these frontier molecular orbital distributions, are attributable to π → π* type transitions. It is clear from figure 6*b* that in the gas phase, the electronic spectra of AI and AI_Al are similar, the only difference being the slight bathochromic effect caused by the doping of the B_12_N_12_ FLN. Indeed, the main absorption bands of these complexes are centred around 271 nm (due to H-5 → L(31%)) and 274 nm (H-7 → L(31%)), respectively, for AI and AI_Al. A strong bathochromic effect can also be observed from figure 6*b* induced by the solvation of these compounds, but their spectra remain similar in aqueous solution. The increments in the values of *λ*_max_ are 11 and 15 nm for AI and AI_Al respectively, upon solvation.

**Table 6.** Absorption maxima (*λ*_max_), absorption energy (*E*), oscillator strength (*F*) and composition of electronic transfer obtained from the electronic spectra of the studied compounds.

		*λ*_max_ (nm)	*E* (eV)	*F*	assignment and composition
Gas	TMZ	193.59	6.40	0.0322	H-4 → L (47%)
		221.95	5.58	0.1161	H → L+1 (44%)
		268.47	4.62	0.2942	H → L (47%)
	AI	218.75	5.67	0.0601	H-5 → L+1 (24%); H-4 → L (11%)
		271.49	4.57	0.3203	H-5 → L (31%); H → L (10%)
		277.11	4.47	0.1362	H → L (36%); H-5 → L (11%)
	CI	221.08	5.61	0.0554	H-3 → L+1 (23%)
		228.07	5.44	0.0796	H → L+1 (30%)
		253.94	4.88	0.1834	H-8 → L (15%); H-3 → L (13%)
		269.91	4.59	0.1558	H-3 → L (30%)
	AI_Al	218.29	5.68	0.0669	H-7 → L+1 (43%)
		274.36	4.52	0.3113	H-7 → L (31%)
		275.79	4.50	0.1723	H-1 → L (31%)
	CI_Al	224.88	5.51	0.0691	H-7 → L+1 (23%); H-2 → L+1 (19%)
		258.53	4.80	0.3111	H-7 → L (32%)
		275.22	4.51	0.0419	H-7 → L (13%)
	AII	311.45	3.98	0.0043	H-13 → L (41%)
		313.60	3.95	0.0060	H → L+1 (29%); H → L (13%)
		314.81	3.94	0.0092	H → L (33%); H → L+1 (12%)
	CII	261.71	4.74	0.2356	H-13 → L (28%)
		277.20	4.47	0.0151	H → L (41%)
		277.76	4.46	0.0485	H-13 → L (8%)
	AII_B	271.44	4.57	0.3644	H-12 → L (23%); H-13 → L (14%)
		328.59	3.77	0.0305	H → L (45%)
	CII_B	266.42	4.65	0.0393	H-1 → L+3 (15%)
		275.36	4.50	0.0780	H → L (33%)
		280.84	4.42	0.0715	H-1 → L (30%)
water	TMZ	197.01	6.29	0.0562	H-4 → L (47%)
		224.05	5.53	0.1443	H → L+1 (45%)
		282.29	4.39	0.3844	H → L (48%)
	AI	205.57	6.03	0.0571	H-11 → L (26%)
		223.22	5.55	0.0958	H → L+1 (44%)
		288.13	4.30	0.5405	H → L (47%)
	CI	273.03	4.54	0.4169	H → L (18%); H-2 → L (16%); H-1 → L (16%)
	AI_Al	207.64	5.97	0.0532	H-11 → L (29%); H-1 → L+2 (16%)
		223.23	5.55	0.0882	H-1 → L+1 (45%)
		290.16	4.27	0.5660	H-1 → L (49%)
	CI_Al	207.80	5.97	0.0533	H-8 → L (26%)
		224.71	5.52	0.1419	H-4 → L (45%)
		273.30	4.54	0.4366	H-3 → L (47%)
	AII	287.83	4.31	0.4756	H-4 → L (40%)
	CII	302.99	4.09	0.0053	H → L+1 (40%)
	AII_B	275.31	4.50	0.0713	H → L (35%)
	CII_B	265.00	4.68	0.0805	H -1 → L (39%)

As concerns the molecular complexes based on Al_12_N_12_ nano-cage, doping also leads to a red shift in both study media. An increment of about 15 nm in the value of *λ*_max_ is observed when passing from AII to AII_B. However, unlike the BN-based complexes studied, a blue shift is observed for the absorption maxima of the Al_12_N_12_-based complexes upon solvation. It should, however, be noticed that the values of excited-state energies as well as the HOMO–LUMO gap of the compounds studied might be improved using range-separated functionals. Indeed, it has been shown that these functionals best describe the mentioned parameters due to their −1.00/R dependence on the exchange potential instead of the −0.50/R dependence of the M06-2X Minnesota functional [48–51].

## 4. Concluding remarks

The adsorption of the anti-cancer drug dubbed TMZ herein at the external surface of pure and doped X_12_N_12_ (X = B and Al) using the DFT/M06-2X-D3/6-311G(d,p) theoretical chemistry method was the main concern of the present work. Quantum chemical calculations were carried out in both gas and aqueous phases. The results have revealed that apart from the doped BAl_11_N_12_ FLN, all the other investigated nano-cages can spontaneously adsorb TMZ in both study media. Moreover, TMZ was found to bind onto the FLNs preferentially through O_12_, via a partially covalent interaction. The complexes involving that O-atom are also found to exhibit the greatest dipole moment values. Charge transfer from TMZ to the respective FLNs was clearly elucidated via NBO and Hirshfeld atomic charge analyses. The analysis of calculated electronic parameters has shown that AI (the most stable molecular complex) witnessed the greatest change in FMOs distribution, *E*_gap_ and *E*_F_. In addition, the release time in vacuum ultraviolet light of AI was found to be only 1.06 s at room temperature. Finally, the best sensor must strongly link the adsorbed molecule (giving rise to negative values of adsorption energy as well as Gibbs free energy), must greatly alter (reduce) the HOMO-LUMO energy gap of the sensor (FLNs herein). The desorption time too must be the smallest possible so that the FLN can be easily regenerated and re-used. So all these parameters are combined to elect the best sensor, the most important being the change in the HOMO-LUMO energy gap as well the desorption time. From the foregoing observations, B_12_N_12_ among the FLNs investigated is the most sensitive to TMZ and can be proposed for industrial applications.

## Data Availability

All output files as well as supplementary materials are available from the Dryad Digital Repository: https://doi.org/10.5061/dryad.6m905qg12 [52].

## References

[1] Zajac A et al. 2021 Involvement of PI3K pathway in glioma cell resistance to temozolomide treatment. Int. J. Mol. Sci. **22**, 5155. (10.3390/ijms22105155) 34068110 PMC8152763

[2] Kizilarslanoglu MC, Aksoy S, Yildirim NO, Ararat E, Sahin I, Altundag K. 2011 Temozolomide-related infections: review of the literature. J. BUON. **16**, 547-550. 22006764

[3] Climans SA et al. 2020 Temozolomide and seizure outcomes in a randomized clinical trial of elderly glioblastoma patients. J. Neurooncol. **149**, 65-71. (10.1007/s11060-020-03573-x) 32632894

[4] Okita Y, Narita Y, Miyakita Y, Ohno M, Aihara K, Mori S, Kayama T, Shibui S. 2012 Reactivation of cytomegalovirus following treatment of malignant glioma with temozolomide. Int. Canc. Conf. J. **1**, 53-57. (10.1007/s13691-011-0009-7)

[5] De Jesus A, Grossman SA, Paun O. 2009 Cytomegalovirus associated colonic pseudotumor: a consequence of iatrogenic immunosuppression in a patient with primary brain tumor receiving radiation and temozolomide. J. Neurooncol. **94**, 445-448. (10.1007/s11060-009-9882-8) 19360378 PMC3696988

[6] Marino A et al. 2009 Multifunctional temozolomide-loaded lipid superparamagnetic nanovectors: dual targeting and disintegration of glioblastoma spheroids by synergic chemotherapy and hyperthermia treatment. Nanoscale **11**, 21 227-21 248. (10.1039/C9NR07976A) PMC6867905 31663592

[7] del Castillo RM, Ramos E, Martínez A. 2021 Interaction of graphene with antipsychotic drugs: is there any charge transfer process? J. Comput. Chem. **42**, 60-65. (10.1002/jcc.26433) 33048373

[8] Zhu J, Lu Z, Jing X, Wang X, Liu Q, Wu L. 2021 Adsorption of temozolomide chemotherapy drug on the pristine BC_3_NT: quantum chemical study. Chem. Pap. **74**, 4525-4531. (10.1007/s11696-020-01232-z)

[9] Silva-Alves DA, Camara MVS, Chaves-Neto AMJ, Gester R, Andrade-Filho T. 2020 Theoretical study of the adsorption of diphenylalanine on pristine graphene. Theor. Chem. Acc. **139**, 83. (10.1007/s00214-020-02594-z)

[10] Sheikhi M, Ahmadi Y, Kaviani S, Shahab S. 2021 Molecular modeling investigation of adsorption of Zolinza drug on surfaces of the B_12_N_12_ and Al_12_N_12_ nanocages. Struct. Chem. **32**, 1181-1196. (10.1007/s11224-020-01697-4)

[11] Noormohammadbeigi M, Kamalinahad S, Izadi F, Adimi M, Ghasemkhan A. 2020 Theoretical investigation of thioguanine isomers anticancer drug adsorption treatment on B_12_N_12_ nanocage. Mater. Res. Express 6, 1250g2. (10.1088/2053-1591/ab672a)

[12] Jabr-Milane LS, van Vlerken LE, Yadav S, Amiji MM. 2008 Multi-functional nanocarriers to overcome tumor drug resistance. Cancer Treat. Rev. **34**, 592-602. (10.1016/j.ctrv.2008.04.003) 18538481 PMC2585991

[13] Mirsa R, Acharya S, Sahoo SK. 2010 Cancer nanotechnology: application of nanotechnology in cancer therapy. Drug Discov. Today **15**, 842-850. (10.1016/j.drudis.2010.08.006) 20727417

[14] Soltani A, Javan MB. 2015 Carbon monoxide interactions with pure and doped _B11_XN_12_ (X = Mg, Ge, Ga) nano-cluster: a theoretical study. RSC Adv. **5**, 90 621-90 631. (10.1039/C5RA12571E)

[15] Jouypazadeh H, Farrokhpour H. 2018 DFT and TD-DFT study of the adsorption and detection of sulfur mustard chemical warfare agent by the C_24_, C_12_Si_12_, Al_12_N_12_, Al_12_P_12_, Be_12_O_12_, B_12_N_12_ and Mg_12_O_12_ nanocages. J. Mol. Struct. **1164**, 227-238. (10.1016/j.molstruc.2018.03.051)

[16] Soltani A, Raz SG, Taghartapeh MR, Moradi AV, Mehrabian RZ. 2013 *Ab initio* study of the NO_2_ and SO_2_ adsorption on Al_12_N_12_nano-cage sensitized with gallium and magnesium. Comput. Mater. Sci. **79**, 795-803. (10.1016/j.commatsci.2013.07.011)

[17] Celaya CA, FelipeHernández-Ayala L, Zamudio FB, Vargas JA, Reina M. 2021 Adsorption of melphalan anticancer drug on C_24_, B_12_N_12_, B_12_C_6_N_6_, B_6_C_12_N_12_ and B_6_C_6_N_12_ nanocages: a comparative DFT study. J. Mol. Liq. **329**, 115528. (10.1016/j.molliq.2021.115528)

[18] Javan MB, Soltani A, Azmoodeh Z, Abdolahi N, Gholami N. 2016 A DFT study on the interaction between 5-fluorouracil and B_12_N_12_ nanocluster. RSC Adv. **6**, 104513. (10.1039/C6RA18196A)

[19] Kian M, Tazikeh-Lemeski E. 2020 B_12_Y_12_(Y: N, P) fullerene-like cages for exemestane-delivery; molecular modeling investigation. J. Mol. Struct. **1217**, 128455. (10.1016/j.molstruc.2020.128455)

[20] Abdolahi N, Aghaei M, Soltani A, Azmoodeh A, Balakheyli H, Heidari F. 2018 Adsorption of celecoxib on B_12_N_12_ fullerene: spectroscopic and DFT/TD-DFT study. Spectrochim. Acta Part A Mol. Biomol. Spectrosc. **204**, 348-353. (10.1016/j.saa.2018.06.077) 29957413

[21] Harris RA. 2019 Chemotherapy drug temozolomide adsorbed onto iron-oxide (Fe_3_O_4_) nanoparticles as nanocarrier: a simulation study. J. Mol. Liq. **288**, 111084. (10.1016/j.molliq.2019.111084)

[22] Oku T, Nishiwaki A, Narita I. 2004 Formation and atomic structure of B12N12nanocage clusters studied by mass spectrometry and cluster calculation. Sci. Technol. Adv. Mater. **5**, 635-638. (10.1016/j.stam.2004.03.017)

[23] Liu C, Hu Z, Wu Q, Wang X, Chen Y, Zhu J. 2006 Controllable synthesis of one-dimensional aluminum nitride nanostructures through vapor-solid epitaxial growth. J. Nanoelectron. Optoelectron. **1**, 114-118. (10.1166/jno.2006.014)

[24] Padash R, Sobhani-Nasab A, Rahimi-Nasrabadi M, Mirmotahari M, Ehrlich H, Rad AS, Peyravi M. 2018 Is it possible to use *X*_12_*Y*_12_ (*X*= Al, B, and *Y*= N, P) nanocages for drug-delivery systems? A DFT study on the adsorption property of 4-aminopyridine drug. Appl. Phys. A Mater. Sci. Process. **124**, 582. (10.1007/s00339-018-1965-y)

[25] Kohn W, Sham LL. 1965 Self-consistent equations including exchange and correlation effects. Phys. Rev. **140**, 1133-1138. (10.1103/PhysRev.140.A1133)

[26] Zhao Y, Schultz NE, Truhlar DG. 2006 Design of density functionals by combining the method of constraint satisfaction with parametrization for thermochemistry, thermochemical kinetics, and noncovalent interactions. J. Chem. Theory Comput. **2**, 364-382. (10.1021/ct0502763) 26626525

[27] Hehre WJ, Ditchfield R, Pople JA. 1972 Self-consistent molecular orbital methods. XIV. An extended Gaussian-type basis for molecular orbital studies of organic molecules. Inclusion of second row elements. J. Chem. Phys. **56**, 5255. (10.1063/1.1677028)

[28] Frisch MJ et al. 2013 Gaussian 09, revision D01. Wallingford, CT: Gaussian, Inc.

[29] Anila S, Suresh CH. 2021 Guanidine as a strong CO_2_ adsorbent: a DFT study on cooperative CO_2_ adsorption. Phys. Chem. Chem. Phys. **23**, 13662. (10.1039/D1CP00754H) 34121106

[30] Al-Otaibi JS, Mary YS, Kaya S, Serdaroglu G. 2021 DFT computational study of trihalogenated aniline derivative's adsorption onto grapheme/fullerene/fullerene-like nanocages, X_12_Y_12_ (X = Al, B, and Y = N,P). J. Biomol. Struct. Dyn. **21**, 1-13. (10.1080/07391102.2021.1914172) 33876711

[31] Aghaei M et al. 2020 Investigations of adsorption behavior and anti-inflammatory activity of glycine functionalized Al_12_N_12_ and Al_12_ON_11_ fullerene-like cages. Spectrochim. Acta Part A Mol. Biomol. Spectrosc. **246**, 119023. (10.1016/j.saa.2020.119023) 33049473

[32] Grimme S, Antony J, Ehrlich S, Krieg H. 2010 A consistent and accurate *ab initio* parametrization of density functional dispersion correction (DFT-D) for the 94 elements H-Pu. J. Chem. Phys. **132**, 154104. (10.1063/1.3382344) 20423165

[33] Lu T, Chen F. 2011 Calculation of molecular orbital composition. Acta Chim. Sin. **69**, 2393-2406.

[34] Marenich AV, Cramer CJ, Truhlar DG. 2009 Universal solvation model based on solute electron density and on a continuum model of the solvent defined by the bulk dielectric constant and atomic surface tensions. J. Phys. Chem. B **113**, 6378-6396. (10.1021/jp810292n) 19366259

[35] Vessally E, Esrafili MD, Nurazar R, Nematollahi P, Bekhradnia A. 2016 A DFT study on electronic and optical properties of aspirin-functionalized B_12_N_12_ fullerene-like nanocluster. Struct. Chem. **28**, 735-748. (10.1007/s11224-016-0858-y)

[36] Sohail M, Khaliq F, Mahmood T, Ayub K, Tabassum S, Gilani MA. 2021 Influence of bi-alkali metals doping over Al_12_N_12_ nanocage on stability and optoelectronic properties: a DFT investigation. Radiat. Phys. Chem. **184**, 109 457-109 468. (10.1016/j.radphyschem.2021.109457)

[37] Khalili A, Baei M, Ghaboos SHH. 2020 Improvement of antioxidative activity of apigenin by B_12_N_12_ nanocluster: antioxidative mechanism analysis electro. Phys. Theor. Chem. **5**, 1829-1836. (10.1002/slct.201904170)

[38] Rad AS, Ayub K. 2016 Ni adsorption on Al_12_P_12_ nano-cage: DFT study. J. Alloy Compd. **678**. 317-324. (10.1016/j.jallcom.2016.03.175)

[39] Padash R, Esfahani MR, Rad AS. 2020 The computational quantum mechanical study of sulfamide drug adsorption onto X_12_Y_12_ fullerene-like nanocages: detailed DFT and QTAIM investigations. J. Biomol. Struct. Dynam. **39**, 5427-5437. (10.1080/07391102.2020.1792991) 32662325

[40] Farrokhpour H, Jouypazadeh H, Sohroforouzani SV. 2019 Interaction of different types of nanocages (Al_12_N_12_, Al_12_P_12_, B_12_N_12_, Be_12_O_12_, Mg_12_O_12_, Si_12_C_12_ and C_24_) with HCN and ClCN: DFT, TD-DFT, QTAIM and NBO calculations. Mol. Phys. **118**, 1626506. (10.1080/00268976.2019.1626506)

[41] Bahrami A, Seidi S, Baheri T, Aghamohammadi M. 2013 A first principles study on the adsorption behavior of amphetamine on pristine, P and Al-doped B_12_N_12_ nanocages. Superlattic. Microstruct. **64**, 265-273. (10.1016/j.spmi.2013.09.034)

[42] Bader RFW. 1990 Atoms in molecules. Ontario, Canada: Oxford University Press.

[43] Popelier PLA. 2000 On the full topology of the Laplacian of the electron density. Coord. Chem. Rev. **197**, 169. (10.1016/S0010-8545(99)00189-7)

[44] Contreras-García J, Boto R, Izquierdo-Ruiz F, Reva I, Woller T, Alonso M. 2016 A benchmark for the non-covalent interaction (NCI) index or… is it really all in the geometry? Theor. Chem. Acc. **135**, 242. (10.1007/s00214-016-1977-7)

[45] Rozas I, Alkorta I, Elguero J. 2000 Behavior of ylides containing N, O and C atoms as hydrogen bond acceprtors. J. Am. Chem. Soc. **122**, 11154. (10.1021/ja0017864)

[46] Weinhold F, Carpenter JE. 1988 The natural bond orbital Lewis structure concept for molecules, radicals, and radical ions. In The structure of small molecules and ions, pp. 227-236. Berlin, Germany: Springer.

[47] Lewis GN. 1916 The atom and the molecule. J. Am. Chem. Soc. **38**, 762-785. (10.1021/ja02261a002)

[48] Wong BM, Hsieh TH. 2010 Optoelectronic and excitonic properties of oligoacenes: substantial improvements from range-separated time-dependent density functional theory. J. Chem. Theory Comput. **6**, 3704. (10.1021/ct100529s) 21170284 PMC3002181

[49] Balanay MP, Kim DH. 2011 Computational study of absorption energies of organic sensitizers used in photovoltaic applications. J. Phys. Chem. C **115**, 19424. (10.1021/jp205512v)

[50] Peach MJG, Tellgren EI, Sałek P, Helgaker T, Tozer DJ. 2007 Structural and electronic properties of polyacetylene and polyyne from hybrid and coulomb-attenuated density functionals. J. Phys. Chem. A **111**, 11930. (10.1021/jp0754839) 17963369

[51] Foster ME, Wong BM. 2012 Nonempirically tuned range-separated DFT accurately predicts both fundamental and excitation gaps in DNA and RNA nucleobases. J. Chem. Theory Comput. **8**, 2682. (10.1021/ct300420f) 22904693 PMC3419459

[52] Ndjopme Wandji BL, Tamafo Fouegue AD, Nkungli NK, Ntieche RA. 2022 Data from: DFT investigation on the application of pure and doped X_12_N_12_ (X = B and Al) fullerene-like nano-cages towards the adsorption of temozolomide. Dryad Digital Repository. (10.5061/dryad.6m905qg12) PMC8992703 35401995

